# Influence of Different Isolation Methods on Chemical Composition and Bioactivities of the Fruit Peel Oil of *Citrus medica* L. var. *sarcodactylis* (Noot.) Swingle

**DOI:** 10.3390/medicines4010001

**Published:** 2017-01-04

**Authors:** Gang Deng, Jonathan D. Craft, Kelly Marie Steinberg, Pei Lei Li, Suraj Kumar Pokharel, William N. Setzer

**Affiliations:** 1 Department of Biotechnology, Zhejiang Normal University, Jinhua 321004, China; peileili93@163.com; 2 Department of Chemistry, University of Alabama in Huntsville, Huntsville, AL 35899, USA; craftjd@gmail.com (J.D.C.); kms0019@uah.edu (K.M.S.); skp0007@uah.edu (S.K.P.); setzerw@uah.edu (W.N.S.)

**Keywords:** vacuum hydrodistillation, ultrafiltration, essential oils, fingered citron, GC-MS analysis, antimicrobial, antioxidant

## Abstract

**Background**: The chemical composition and bioactivities of essential oils (EOs) of fingered citron (*Citrus medica* L. var. *sarcodactylis* (Noot.) Swingle) are considerably sensitive and lapsible during high-temperature processing of traditional separating techniques. In the present research, vacuum distillation and ultrafiltration were utilized in order to process the concentrated juice from fingered citron, obtaining a high-quality essential oil. **Methods**: In order to compare the essential oils obtained by conventional means, the chemical compositions of the essential oils were analyzed using GC-MS, before antimicrobial and antioxidant screening assays were carried out. **Results**: Oil which had been subjected to vacuum distillation was shown to maintain most of the distinctiveness of the fingered citron, due to its high content of characteristic flavor components and low content of cyclic oxygenated monoterpenoids. Interestingly, the oil obtained by ultrafiltration showed notable in vitro antimicrobial activity. The DPPH· radical-scavenging assay method revealed that the antioxidant abilities were as follows, presented in descending order: vacuum distillation oil > hydrodistillation oil > ultrafiltration oil. **Conclusions**: The essential oil obtained by vacuum distillation could be combined with the juice produced from fingered citron to create one of the most promising techniques in the fine-processing of citron fruits.

## 1. Introduction

*Citrus medica* L. var. *sarcodactylis* (Noot.) Swingle, commonly known as fingered citron in China, is an uncommon variation of *Citrus medica* L., belonging to the Rutaceae family [1,2]. It is well-known as a precious Chinese traditional medicine and is widely cultivated from the central area of China, to the southeastern coast, especially in the Jinhua district of the Zhejiang province, lying to the south of Shanghai [3]. The fruits are widely manufactured for use in various types of processed foods, such as healthcare tea, drink flavoring, succade, and traditional desserts. Also, the intact fresh fruits could be directly sold or used as decorative bonsai at international markets, extending from Asia to South America, mostly due to their unique finger-like appearance and exquisite aroma. However, recently, due to the requirement for the modernization of traditional Chinese herbs, attention has been focused on research relating to bioactive components and the development of advanced processing techniques for fingered citron. In particular, as both a medicinal and edible product, it has been acknowledged that the essential oil of fingered citron is a major component of the commercial sector, with an extensive use as a flavoring agent for beverages, ice cream [4], biscuits, cakes, healthcare food [5], and household products [6,7]. Therefore, in terms of commercialization of fingered citron, it is of utmost importance that we obtain a high-quality essential oil, at the lowest cost possible, during the processing of the fruits.

Unlike other citrus fruits such as lemon and grapefruit, mature fingered citron has only a thin yellow exocarp and thick white entoderm, without juicy pulp or seeds [2]. This peculiar anatomical structure, results in a low proportion of exocarp containing essential oil. Therefore, the total yield of a complete fruit is typically in the range of 0.7%–1.2% (*w*/*w*), as stated in previously published work [8,9]. Furthermore, the oil-water state is sometimes impossible to separate, depending on single-circle hydrodistillation, due to the emulsification of a much lower essential oil content into a relatively large amount of water. Therefore, a conventional isolation process for producing essential oil from these fruits usually employs hydrodistillation using condensed fluid, repeatedly returning to the evaporator-like Clevenger apparatus. However, for a practical large-scale production of essential oil, the cost of isolating the essential oil needs to be considered, and the essential oil could be combined with other procedures, such as beverage production. Much research has focused on oil separation following the cold-pressing of citrus fruits, with pulp such as the common orange [10], but few investigations have pursued using fingered citrons, which have no pulp.

The quality of essential oils produced from fingered citron is highly dependent on its chemical composition, as well as associated bioactivities [11]. During the course of processing the peels of mature fruits, different isolation techniques can not only result in different oil yields, but can also have noticeable effects on chemical composition and other related oil qualities [12,13]. Therefore, owing to potential commercial applications, there have been several investigations that have attempted to demonstrate the composition and stability of characteristic aromas, and to determine possible bioactivities, such as those which are antimicrobial or antioxidant [14,15]. The GC-MS analysis of oils from Japanese fingered citron showed that β-ionone, one of the aroma components with a very low odor threshold (7 ng/L), was responsible for the characteristic osmanthus-like aroma of the fruit [16]. However, more recent studies have determined the characteristic aromatic components of essential oils from the Jinhua district of China, the results of which, using gas chromatography-olfactometry (GC-O), have demonstrated that α-pinene and α-bergamotene were the principal contributors to the characteristic aroma of fingered citron oil [17]. In addition, as a traditional Chinese medicinal plant, fingered citron has usually been employed as a stomachic carminative, asthma expectorant, and anxiolytic [1,9]. Although an increasing number of oil bioactivities, including hypotensive and hypolipidemic effects, have been confirmed [5], there have been no reports on the effects of isolation procedures on the chemical composition and bioactivities.

This work presents two innovative isolation techniques for directly separating the essential oils from the juices of fingered citron. It is important to note that both of the methodologies proposed here do not represent conventional solid-water mixing distillation of essential oils, but a new separation method of the concentrated upper layer of fruit juice. One is a short-time vacuum distillation, and the other is ultrafiltration. The data reported in this work were obtained by comparing the chemical compositions and bioactivities of fingered citron oils using the new methods, with those of the oils obtained by conventional methods such as hydrodistillation and organic solvent extraction. The purpose of this research is to obtain a high-quality, commercially applicable fingered citron oil, that can maintain the pure distinctiveness of fingered citron. It is very likely that improving the quality of fingered citron oil, as well as reducing the processing cost, would significantly improve local agricultural production.

## 2. Materials and Methods

### 2.1. Materials

**Figure 1:**
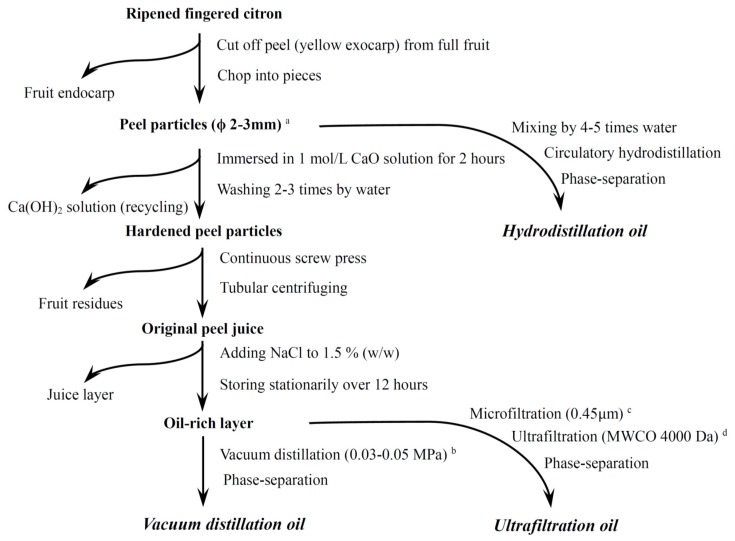
Scheme showing different isolation methods of essential oil from fingered citron fruits. ^a^ The average diameter of fruit peel particle is around 2–3 mm; ^b^ The absolute pressure in the vacuum is 0.03–0.05 MPa (absolute pressure); ^c^ The pore diameter of microfiltration is 0.45 μm; ^d^ The molecular weight cut-off (MWCO) of ultrafiltration is 4000 Da.

The fresh fruits of *Citrus medica* L. var. *sarcodactylis* Swingle were harvested in November 2014, from Jinhua (29.11° N, 119.64° E, 55 m above sea level), Zhejiang, China. The essential oil isolation procedures of fingered citron were performed as presented in Figure 1. The vacuum distillation was maintained at 65–85 °C, according to the distillate flow rate. The ultrafitrating operation was completed at room temperature, and the filtration rate was only 17.5–21 mL/h. The yields of vacuum distillation oil, ultrafiltration oil, and hydrodistillation oil, were 0.73% ± 0.12%, 0.28% ± 0.09%, and 1.07% ± 0.14%, respectively.

### 2.2. Gas Chromatographic/Mass Spectral Analysis

The essential oils of fingered citron were analyzed by GC-MS [18], using an Agilent 6890 GC (Agilent Technologies, Palo Alto, CA, USA) with an Agilent 5973 mass selective detector (Agilent Technologies, Palo Alto, CA, USA, operated in the EI mode (electron energy = 70 eV), scan range = 40–400 mAU, and scan rate = 3.99 scans/s), and an Agilent ChemStation data system (G1701CA, Agilent Technologies, Palo Alto, CA, USA). The GC column was a HP-5ms fused silica capillary with a (5% phenyl)-polymethylsiloxane stationary phase, a film thickness of 0.25 μm, a length of 30 m, and an internal diameter of 0.25 mm. The carrier gas was helium, with a column head pressure of 48.7 kPa and a flow rate of 1.0 mL/min. Injector temperature was 200 °C and interface temperature was 280 °C. The GC oven temperature program was used as follows: 40 °C initial temperature, held for 10 min; increased at 3 °C/min to 200 °C; increased 2 °C/min to 220 °C. A 1% *w*/*v* solution of the sample in CH_2_Cl_2_ was prepared and 1 μL was injected using a splitless injection technique.

Identification of the oil components was based on their retention indices, determined by the reference to a homologous series of *n*-alkanes, and by a comparison of their mass spectral fragmentation patterns with those reported in the literature [19] and stored on the MS library (NIST database (Version G1036A, Revision D.01.00, US Department of Commerce, Gaithersburg, MD, USA)/ChemStation data system (Version G1701CA, Revision C.00.01.080, Agilent Technologies, Palo Alto, CA, USA)). The percentages of each component are reported as raw percentages, based on total ion current without standardization.

### 2.3. Antimicrobial Screening

The essential oils were screened for antibacterial activity against: Gram-positive bacteria, *Staphylococcus aureus* (ATCC No. 29213), *Bacillus cereus* (ATCC No. 14579); Gram-negative bacteria *Pseudomonas aeruginosa* (ATCC No. 27853), *Serratia marcescens* (ATCC No. 14756). Minimum inhibitory concentrations (MIC) were determined by using the microbroth dilution technique [20]. Dilutions of the essential oil were prepared in cation-adjusted Mueller Hinton broth (CAMHB), beginning with 50 μL of 1% *w*/*w* solutions of essential oil in DMSO, plus 50 μL CAMHB. The extract solutions were serially diluted (1:1) in CAMHB, in 96-well plates. Organisms at a concentration of approximately 1.5 × 10^8^ colony forming units (CFU)/mL were added to each well. Plates were incubated at 37 °C for 24 h; the final minimum inhibitory concentration (MIC) was determined as the lowest concentration without turbidity. Gentamycin was used as a positive antibiotic control; DMSO was used as a negative control. Antifungal activity against *Candida albicans* (ATCC No. 10231) and *Aspergillus niger* (ATCC No. 16888) was determined as above, using Yeast and Mold Broth (YMB) inoculated with the corresponding fungal culture, diluted to a McFarland turbidity of 1.0. Amphotericin B was the positive control [18].

### 2.4. Antioxidant Activity Screening

The samples were also subjected to screening in order to identify their possible antioxidant activity, by using a 1,1-diphenyl-2-picrylhydrazyl free radical (DPPH·) scavenging assay [21]. Free radical scavenging activity of the corresponding samples was dependent on the extent of bleaching of the purple-colored DPPH methanol solution. Aliquots (50 μL) of various concentrations (0.5–3.0 mg/mL) of the samples in methanol were added to 5 mL of 0.004% DPPH solution in methanol (5 mL). Following a 30-min incubation period at room temperature, the absorbance was recorded against a blank at 517 nm. Inhibition of DPPH· in percentage (I%) was calculated using the following formula:

(1) $$I_{DPPH}\%=\left(1-\frac{A_{0}}{A_{x}}\right)\times 100$$

where *A*_0_ is the absorbance of the control reaction (containing all reagents except the test compound), and *A_x_* is the absorbance of the test sample. The median inhibitory concentration (IC_50_) was calculated from the plot of inhibition percentage, against sample concentration. Tests were carried out in triplicate.

## 3. Results

### 3.1. Essential Oil Compositions

The essential oils obtained by the different isolation procedures are characterized by the presence of cyclic and acyclic monoterpenes, which may or may not possess oxygen atoms in their structures (Table 1). All of oil components are divided into eight groups, including cyclic monoterpene hydrocarbons (I), acyclic monoterpene hydrocarbons (II), acyclic oxygenated monoterpenoids (III), aldehydes (IV), cyclic oxygenated monoterpenoids (V), sesquiterpene hydrocarbons (VI), oxygenated sesquiterpenoids (VII), and coumarins (VIII).

### 3.2. Antimicrobial Activity of Fingered Citron Oils

The MICs of three fingered citron oils against Gram-positive bacteria, Gram-negative bacteria, and fungi, are reported in Table 2. In particular, the ultrafiltration oil showed notable in vitro antimicrobial activity against *Staphylococcus aureus* (MIC = 156 μg/mL), *Bacillus cereus* (MIC = 156 μg/mL), *Pseudomonas aeruginosa* (MIC = 39 μg/mL), and *Aspergillus niger* (MIC = 20 μg/mL).The vacuum distillation oil was only marginally active against *Staphylococcus aureus* (MIC = 625 μg/mL), *Bacillus cereus* (MIC = 313 μg/mL), and *Pseudomonas aeruginosa* (MIC = 625 μg/mL), but was also strongly active against *Aspergillus niger* (MIC = 156 μg/mL).

### 3.3. Antioxidant Activity

**Figure 2:**
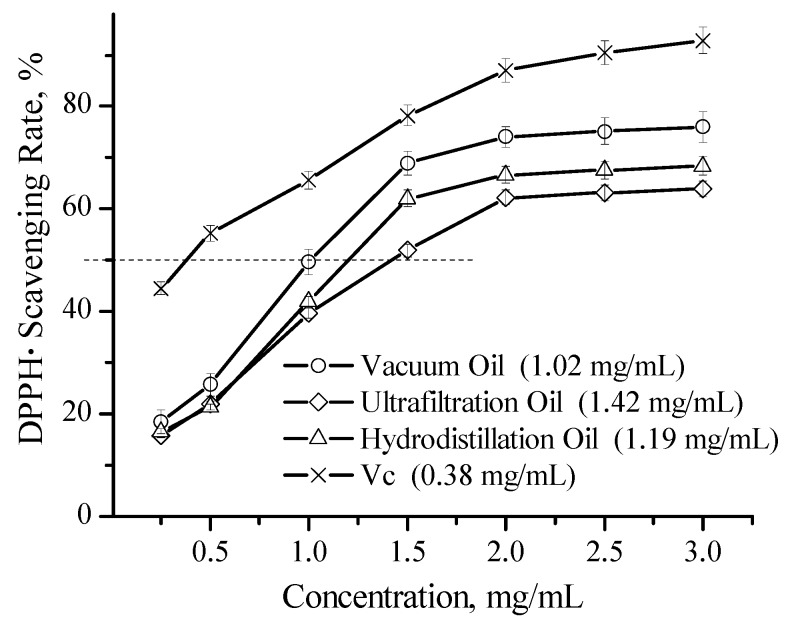
DPPH· scavenging effectiveness of tree oils and ascorbic acid (Vc). Data are expressed as means ± SD of three experiments.

The antioxidant activity of essential oils is another biological property of great interest for potential utilization in cosmetic supplements or functional food products. The use of a DPPH· scavenging assay is the most routinely practiced method for the assessment of anti-radical properties of different samples. As shown in Figure 2, the antioxidant efficiency of the tested essential oils primarily relies on their concentration, and their free radical scavenging effectiveness was as follows, given in descending order and based on the increasing median inhibitory concentration (IC_50_) values: vacuum distillation oil (1.02 mg/mL) > hydrodistillation oil (1.19 mg/mL) > ultrafiltration oil (1.42 mg/mL).

## 4. Discussions

**Figure 3:**
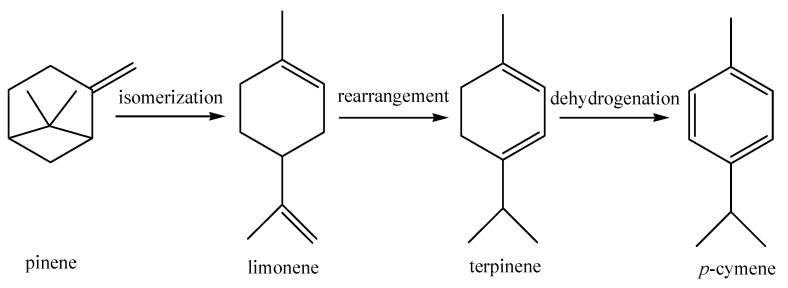
Potential conversion of pinene to *p*-cymene.

The cyclic monoterpenes (I) not containing oxygen atoms are common components in the essential oils of most citrus fruit peels. Previous work has suggested that the mechanism for the formation of *p*-cymene occurs during a rearrangement reaction in which limonene rearranges to form terpinenes, which then dehydrogenates to form *p*-cymene, while pinene and limonene can interconvert with each other through isomerization (Figure 3) [22]. Oils obtained using three different methods have no significant differences in their pinene (α- or β-) content. However, when only focusing on the three most abundant compounds (limonene, γ-terpinene, and *p*-cymene), the oil obtained by vacuum distillation possessed a relatively low content of limonene (47.6%), a relatively high content of γ-terpinene (29.5%), and an absence of *p*-cymene, while the oil obtained by hydrodistillation revealed limonene (60.1%), γ-terpinene (6.1%), and *p*-cymene (17.6%). Notably, the oil produced from ultrafiltration had the highest limonene (64.7%) and *p*-cymene content (26.1%), and the lowest γ-terpinene content (0.1%). The differences in content between these three compounds are most likely related to the considerable interconversion which occurs during the isolation process (Figure 1). The significantly different isolation conditions, including pressure, temperature, and time, seems to have largely influenced the rates of interconversion. For example, ultrafiltration is a slow procedure with an outflow speed of only 20–30 mL/h, so it was necessary to complete this at relatively high pressures (0.15–0.18 MPa) for long periods of time (10–15 h), while vacuum distillation is almost complete within 0.5–1 h, at a pressure of 0.03–0.05 MPa and a temperature of 80–85 °C.

Generally, the diagnostic index for good quality citrus oils largely depends on the quantities of two types of compounds; acyclic monoterpene hydrocarbons (II) and acyclic oxygenated monoterpenoids (III) [23]. By comparing the relative ratio (RR) in Table 3, of either quantity of compounds or their contents, the value of oil from vacuum distillation is of a much higher quality than the oils produced from the other two procedures. Conversely, the concentration of oxygenated terpenoids, including cyclic oxygenated monoterpenoids (V) and oxygenated sesquiterpenoids (VII), can indicate the likelihood that oils have been exposed to the air or light for long periods during isolation or storage. These two classes of compounds can sometimes result in a few unpleasant odors, which will have negative consequences for the oil quality. As shown in Table 3, the relative ratios of cyclic oxygenated monoterpenoids are 8.7 (ultrafiltration), 2.3 (hydrodistillation), and 3.8 (organic solvent extraction) [16], respectively. Low concentrations of the cyclic oxygenated monoterpenoids and the absence of oxygenated sesquiterpenoids, are also characteristic of the oil isolated by vacuum distillation. Relatively quick operation and vacuum conditions minimize the time that the oils are exposed to air and considerably high temperatures, thus achieving high quality oils.

It is of utmost importance that the oils obtained retain the distinctive aroma compounds of the fingered citron fruit. β-Ionone, one of the oxygenated sesquiterpenoids, was thought to be the most characteristic aroma component in the peel of fingered citron [16], but it was not detected in the oils from Zhejiang, China, using three different isolation methods. More recently, gas chromatography-olfactometry (GC-O) has been used to analyze the main odorous compounds in fingered citron essential oil [17]. The results demonstrated that α-pinene and α-bergamotene were the key compounds contributing to the characteristic aroma of fingered citron oil. In this research, the content of α-bergamotene in vacuum distilled oil was greater than that in oils obtained from ultrafiltration or hydrodistillation, while the concentrations of α-pinene in the three types of oils were very similar. These results can help to explain the intensity of the aroma of the oil produced using vacuum distillation.

When analysing conventional hydrodistillation oil, either antibacterial activity or antifungal activity was extremely weak. In previous work, the oils from conventional hydrodistillation had shown weak antibacterial activity in zone-of-inhibition assays, that was partly consistent with the present results, but antifungal inhibitory concentrations were not determined [24]. The antimicrobial activity of the ultrafiltration oil can be attributed to the high concentration of *p*-cymene. This compound has shown antibacterial activity against *Bacillus cereus* [25] and *Escherichia coli* [26], as well as antifungal activity against several *Candida* spp. [27] and filamentous fungi [28].

The different antioxidant activities can be mainly ascribed to their complex compositions, which have different chemical profiles. Since an essential oil is not a pure single compound, its activities often refer to the actions of inter-components, such as synergism and antagonism, which are rarely experimentally supported, thus remaining speculative. However, they can be used to explain the observed bioactivities [14]. Isolation methods are generally believed to result in a variation of naturally occurring antioxidants in the raw materials of a plant. Intense thermal treatment or prolonged air-exposure may be responsible for the oxygenated reactions of monoterpenes or sesquiterpenes in fingered citron oils. Extensive formation of oxygenated products might have significant negative effects on their free radical scavenging effectiveness. More specifically, even when ultrafiltration is carried out at room temperature (20–25 °C), the resulting oils showed lower free radical-scavenging activity, in addition to a marked loss of monoterpene or sesquiterpene hydrocarbons, and an evident increase of oxygenated terpenoids.

## 5. Conclusions

In this work, the influence of different isolation methods on the chemical composition and bioactivities of the fruit peel oil from fingered citron were studied in detail. The various isolation methods included significantly different pressures, temperatures, and duration times, which have the potential to affect the reaction rates of interconversion, such as rearrangement, isomerization, and dehydrogenation. Compared to the oils obtained from other isolation methods, the vacuum distillation oil possessed a comparatively high ability to maintain more characteristic components and fewer oxygenated products (cyclic oxygenated monoterpenoids). In addition, the bioactivity screening assays showed that the ultrafiltration oil displayed notable in vitro antimicrobial activity against some bacteria and mold, while vacuum distillation oil had a relatively high antioxidant activity. In sum, the vacuum distillation method, combined with the juice production of high-quality fingered citron oil, could be thought of as one of the most promising techniques for the fine-processing of citron fruits.

## Figures and Tables

**Table 1:** Peak Identification and Weight Percentage for the Components of Essential Oils.

Entry	Compounds	KI ^a^	VD Oil ^c^		UF Oil ^d^		HD Oil ^e^		Ref Oil ^f^	
			RI ^b^	Area (%)	RI	Area (%)	RI	Area (%)	RI	Area (%)
(I) cyclic monoterpene hydrocarbons										
1	α-thujene	930	931	0.649	932	0.192	933	0.552	924	1.34
2	α-pinene	939	936	3.317	938	2.440	938	3.721	934	2.92
3	camphene	954							944	0.03
4	sabinene	975	974	0.143			974	0.065	967	0.40
5	β-pinene	979	976	1.887	978	1.793	976	1.952	975	2.70
6	α-phellandrene	1002	1003	0.264					999	0.09
7	α-terpinene	1017	1016	0.688					1012	0.68
8	*p*-cymene	1024			1023	26.099	1026	17.561	1015	0.13
9	limonene	1029	1035	47.599	1029	64.667	1031	60.129	1022	47.79
10	γ-terpinene	1059	1064	29.455	1058	0.050	1058	6.172	1049	32.08
11	terpinolene	1088	1089	1.651			1087	0.154	1084	1.37
12	*p*-cymenene	1091			1090	0.072				
(II) acyclic monoterpene hydrocarbons										
13	β-myrcene	990	992	1.551	991	0.653	991	1.786	982	1.67
14	(*Z*)-β-ocimene	1037	1040	5.914			1038	5.098	1031	0.36
15	(*E*)-β-ocimene	1050	1050	1.377			1048	0.601	1039	0.52
16	*allo*-ocimene	1132	1130	0.345						
(III) acyclic oxygenated monoterpenoids										
17	linalool	1096	1101	0.113					1087	0.15
18	citronellal	1153	1153	0.055					1132	0.17
19	nerol	1229	1227	0.123						
20	(*Z*)-ocimenone	1229			1228	0.273				
21	neral	1238	1240	0.706					1216	0.38
22	geraniol	1252	1253	0.093					1219	1.56
23	linalyl acetate	1257							1210	0.10
24	geranial	1267	1269	0.945					1238	0.45
25	neryl acetate	1361	1364	0.018	1364	0.022	1364	0.059	1341	0.04
26	geranyl acetate	1381	1384	0.018	1385	0.029	1385	0.103	1359	0.05
(IV) aldehydes										
27	nonanal	1150							1083	0.02
28	decanal	1201	1200	0.014					1187	0.00
(V) cyclic oxygenated monoterpenoids										
29	*cis*-limonene oxide	1136							1119	0.02
30	*trans*-limonene oxide	1142								
31	terpinen-4-ol	1177	1173	0.121	1177	0.185	1176	0.071	1166	0.13
32	*p*-cymen-8-ol	1182			1185	0.266	1184	0.077		
33	α-terpineol	1188	1185	0.091	1190	0.076	1190	0.055	1177	0.67
34	*trans*-carveol	1216			1217	0.284	1217	0.129		
35	*cis*-carveol	1229					1228	0.099		
36	carvone	1243			1240	0.786	1241	0.063		
37	carvyl acetate	1282			1279	0.234				
(VI) sesquiterpene hydrocarbons										
38	δ-elemene	1338	1335	0.026						
39	α-cubebene	1348	1347	0.014						
40	β-cubebene	1388	1388	0.007						
41	β-elemene	1390	1390	0.010						
42	α-*cis*-bergamotene	1412	1413	0.027	1414	0.016	1413	0.020		
43	β-caryophyllene	1419	1416	0.488			1416	0.012	1425	0.13
44	α-trans-bergamotene	1434	1434	0.453	1434	0.300	1433	0.387	1436	0.13
45	γ-elemene	1436							1498	0.08
46	α-humulene	1454	1451	0.041						
47	β-*trans*-farnesene	1456	1457	0.044			1455	0.030		
48	germacrene D	1485	1479	0.519	1480	0.012	1481	0.017	1484	0.28
49	β-ionone	1488							1468	0.03
50	bicyclogermacrene	1500	1495	0.108						
51	α-bisabolene	1503	1503	0.044			1501	0.020		
52	β-bisabolene	1505	1507	0.510	1503	0.429	1507	0.556	1503	0.21
52	δ-cadinene	1523	1523	0.016						
(VII) oxygenated sesquiterpenoids										
54	caryophyllene oxide	1583			1585	0.132	1585	0.260		
(VIII) coumarins										
55	5,7-dimethoxycoumarin	1937							1934	0.27
	Total			99.442		99.011		99.749		96.951

^a^ Kovats index (KI) on DB-5 in reference to *n*-alkanes [19]; ^b^ Experimental retention index on a HP-5 ms column; ^c^ Oils obtained from vacuum distillation of the top layer of cold-pressed juice; ^d^ Oils obtained from ultrafiltration of the top layer of cold-pressed juice; ^e^ Oils obtained from conventional hydrodistillation of fruit peels; ^f^ Oils extracted by using organic solvent (*n*-pentane and dichloromethane, 1/1 *wt*/*wt*) by Shiota [16].

**Table 2:** Antimicrobial activity of fingered citron peel oils.

Sample	Antimicrobial Activity (MIC, μg/mL)					
	*S. aureus* (G+) ^a^	*B. cereus* (G+)	*P. aeruginosa* (G−) ^b^	*S. marcescens* (G−)	*C. albicans* (Fungus)	*A. niger* (Fungus)
Vacuum distillation oil	625	313	625	1250	2500	156
Ultrafiltration oil	156	156	39	625	1250	20
Hydrodistillation oil	1250	1250	1250	1250	2500	625

^a^ G+ is short for Gram-positive; ^b^ G− is short for Gram-negative.

**Table 3:** Summary Analysis of the Classes of Components in the Essential Oils.

No.	Compounds	VD Oil			UF Oil			HD Oil			Ref Oil		
		Count ^a^	STC ^b^ (%)	RR ^c^	Count	STC (%)	RR	Count	STC (%)	RR	Count	STC (%)	RR
(I)	cyclic monoterpene hydrocarbons	9	85.654	1	7	95.313	1.113	8	90.305	1.054	11	89.53	1.045
(II)	acyclic monoterpene hydrocarbons	4	9.187	1	1	0.653	0.071	3	7.485	0.815	3	2.55	0.278
(III)	acyclic oxygenated monoterpenoids	8	2.071	1	3	0.324	0.156	2	0.162	0.078	8	2.90	1.400
(IV)	aldehydes	1	0.014	1	0	0	0	0	0	0	2	0.02	1.492
(V)	cyclic oxygenated monoterpenoids	2	0.211	1	6	1.832	8.682	6	0.493	2.335	3	0.82	3.886
(VI)	Sesquiterpene hydrocarbons	14	2.306	1	4	0.757	0.328	7	1.044	0.453	6	0.86	0.373
(VII)	Oxygenated sesquiterpenoids	0	0	-	1	0.132	-	1	0.260	-	0	0	-
(VIII)	coumarins	0	0	-	0	0	-	0	0	-	1	0.27	-
Sum of Identified Compounds		38			22			27			34		
Featuring Aroma Intensity		very intense			light			medium intense			not given		

^a^ subtotal number for each type of compound; ^b^ subtotal content for each type of compound; ^c^ relative ratio (RR) to subtotal content of VD oil.
